# Genomic Research as a Critical Pathway to Diagnosis for Individuals with Rare Disease

**DOI:** 10.64898/2026.09.15.26363084

**Published:** 2026-09-20

**Authors:** Anne O'Donnell-Luria, Stephanie DiTroia, Melanie C. OLeary, Lynn Pais, Vijay S. Ganesh, Gabrielle Lemire, Emily OHeir, Ashana Neale, Alicia Pham, Grace E. VanNoy, Brian E. Mangilog, Moriel Singer-Berk, Alba Sanchis-Juan, Hana Snow, Siwaar Abouhala, Stacey Agu, Mutaz Amin, Samantha M. Baxter, Benjamin Blankenmeister, Kinga M. Bujakowska, Colleen M. Carlston, Sanaa Choufani, Laura E. Covill, Eleina M. England, Carmen Glaze, Julia Goodrich, Emily Groopman, Kristen Laricchia, Alysia Kern Lovgren, Jialan Ma, Eva Martinez, Chloe Mighton, Briana OLeary, Ikeoluwa A. Osei-Owusu, Sander Pajusalu, Lindsay Romo, Kathryn Russell, Riccardo Sangermano, Eleanor Seaby, Jillian Serrano, Gulalai Shah, Mugdha Singh, Kayla M. Socarras, Sarah L. Stenton, Miriam S. Udler, Ben Weisburd, Rosanna Weksberg, Clara E. Williamson, Rare Genomes Project, Niall Lennon, Christina Austin-Tse, Harrison Brand, Michael E. Talkowski, Daniel MacArthur, Monica H. Wojcik, Heidi L. Rehm

## Abstract

Many individuals with rare monogenic disease remain molecularly undiagnosed due to challenges accessing genetic testing, ambiguity in interpretation of uncertain variants, and latency between novel disease-gene discovery and adoption into clinical pipelines. The Rare Genomes Project (RGP) provides a remote, research-based genomic sequencing (GS) service for rare disease families to overcome diagnostic barriers while advancing the understanding of the genetic basis of rare disease. Short-read GS and family-centered analysis were performed to identify causes of primary disease. Variants meeting return criteria were clinically confirmed and returned to families. Since 2017, RGP enrolled clinically heterogeneous, undiagnosed rare disease families from all fifty states, Washington DC, and Puerto Rico. Findings were returned to 272 of 1014 sequenced families, resulting in a solve rate of 23.3% and a returned strong candidate rate of 3.5%. Condition-specific return rates ranged from 15% to 40% with many families receiving a result in a known disease gene after healthcare access challenges. Research interventions contributed to returns in 16% of all families and included variants in recently discovered or non-coding disease genes, additional functional studies to resolve variant uncertainty, and real-time data sharing to empower novel disease-gene discovery. RGP demonstrates that a research-based, patient-driven remote sequencing study can effectively bridge a diagnostic gap for rare disease families underserved in current clinical care. This study also underscores the clinical utility of GS with frequent re-analysis and highlights the critical continued role of research in variant interpretation, method innovation, and genomic knowledge base expansion for rare disease diagnosis.

## Full Text Availability

The license terms selected by the author(s) for this preprint version do not permit archiving in PMC. The full text is available from the preprint server.

